# A Case of Intractable Lung Abscess Following Dropped Gallstone-Induced Subphrenic Abscess: A Rare Postoperative Complication Caused by Dropped Gallstone During Laparoscopic Cholecystectomy

**DOI:** 10.7759/cureus.27491

**Published:** 2022-07-30

**Authors:** Keiji Nagata, Takahisa Fujikawa, Soichi Oka, Toshihiro Osaki

**Affiliations:** 1 Surgery, Kokura Memorial Hospital, Kitakyushu, JPN; 2 Thoracic Surgery, Kokura Memorial Hospital, KItakyushu, JPN; 3 Thoracic Surgery, Kokura Memorial Hospital, Kitakyushu, JPN

**Keywords:** residual gallstone, dropped gallstone, laparoscopic cholecystectomy, lung abscess, subphrenic abscess

## Abstract

Dropped gallstones into the abdominal cavity due to perforation of the gallbladder occasionally occur during laparoscopic cholecystectomy. Abscess formation caused by residual gallstones is one of the late postoperative complications after laparoscopic cholecystectomy. Most of them are intra-abdominal abscesses; however formation of intra-thoracic abscesses, in particular, lung abscess, is less described, and surgery for an intra-thoracic abscess is rarely performed. We describe a case of intractable lung abscess following dropped gallstone-induced subphrenic abscess caused by a residual gallstone after laparoscopic cholecystectomy.

## Introduction

Perforation of the gallbladder and spillage of gallstones into the abdominal cavity occasionally occurs during laparoscopic cholecystectomy. Surgeons attempt to extract these dropped gallstones. However, gallstones may become fragmented, inaccessible, or overlooked, leaving gallstones within the intra-abdominal cavity. Abscess formation caused by residual gallstones is one of the late postoperative complications after laparoscopic cholecystectomy [1-4]. Even though most of them are in the abdomen, an intra-thoracic abscess is rare, and so is surgery for intra-thoracic lesions.

We present a case of lung abscess following subphrenic abscess caused by a residual gallstone after laparoscopic cholecystectomy. We provide a review of the literature on dropped gallstones during cholecystectomy.

## Case presentation

**Figure 1:**
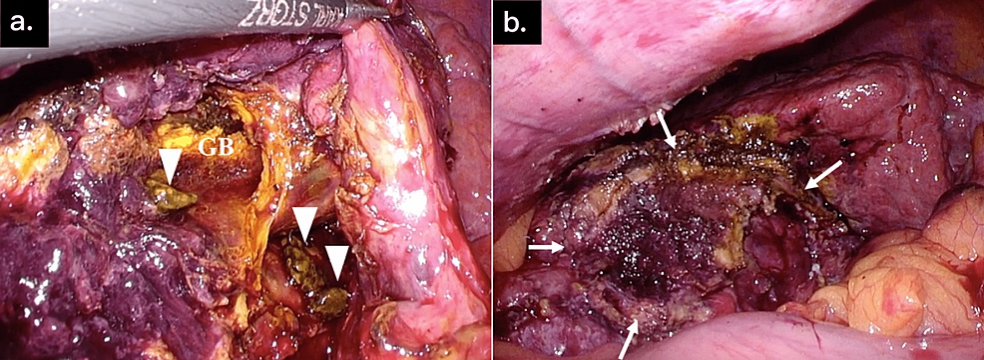
Intra-abdominal findings during laparoscopic cholecystectomy (a) Inflammation of the gallbladder was extremely severe, and a few gallstones were dropped into the abdominal cavity (arrowheads) during laparoscopic cholecystectomy. (b) Laparoscopic cholecystectomy was performed (arrows). GB - gallbladder

A 73-year-old male with acute myocardial infarction and type 2 diabetes mellitus was treated with percutaneous transhepatic gallbladder aspiration (PTGBA) and antibiotic therapy for acute cholecystitis. One month later, elective laparoscopic cholecystectomy was scheduled. Inflammation of the gallbladder was extremely severe, the gallbladder wall was injured, and a few gallstones were dropped into the abdominal cavity during the laparoscopic cholecystectomy, most of which were retrieved (Figure 1a). Finally, laparoscopic cholecystectomy was performed for this severe cholecystitis (Figure 1b). Profuse lavage was carried out, and a closed suction drain was left in the Morrison's pouch. A postoperative antibiotic (sulbactam/cefoperazone) was administered for 11 days. The postoperative course was uneventful, and the patient was discharged on postoperative day 13. The patient came back a week later for follow-up care after surgery. At that time, he had no symptoms at all.

**Figure 2:**
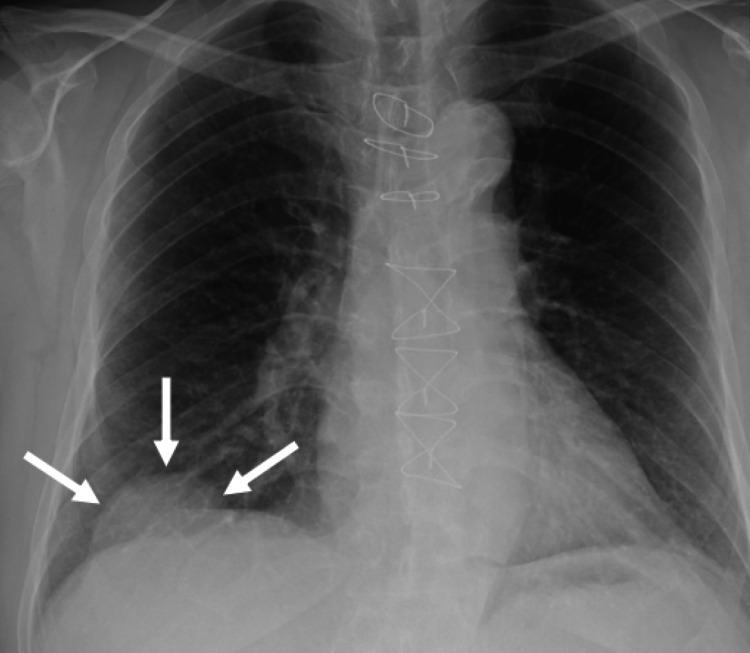
A chest X-ray finding six months after the initial surgery A chest X-ray, six months after initial surgery, showed an abnormal shadow protruding from the hepatic dome (arrows).

**Figure 3:**
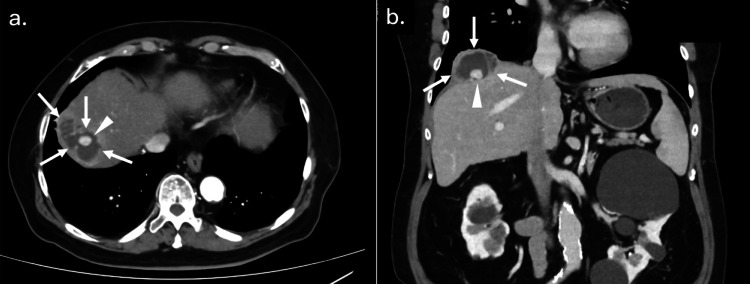
Abdominal CT scan findings six months after the initial surgery (a, b) Abdominal CT, six months after laparoscopic cholecystectomy, revealed an encapsulated fluid retention (arrows) compatible with an abscess containing a calcified lesion (arrowheads) in the right subphrenic space.

Six months after surgery, a chest X-ray taken for a medical check-up showed an abnormal shadow protruding from the hepatic dome (Figure 2). Abdominal computed tomography (CT) was performed for further examination. Although he had no symptoms, CT revealed an encapsulated fluid retention compatible with an abscess containing a calcified lesion in the right subphrenic space (Figure 3).

**Figure 4:**
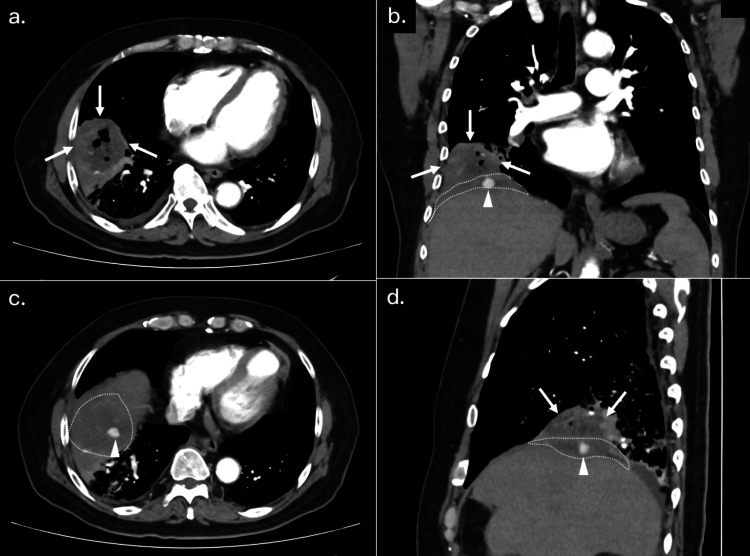
CT scan findings when hemoptysis appeared. (a-d) A contrast-enhanced CT, when the patient presented with fever, wet cough, and hemoptysis, revealed a 70 x 75 mm lung abscess (arrows) and pneumonia in the right lower lobe. The residual gallstone (arrowheads) was in the subphrenic abscess cavity (dotted areas).

Since he did not show any symptoms at all, we did not give any treatment at the time of the first CT, and we planned a short-term follow-up. However, one week after this CT examination, the patient presented to the emergency department with complaints of fever, right chest pain, wet cough, and hemoptysis. He had no abdominal symptoms. Laboratory studies were significant for an elevated white blood cell count of 9.3×10^3^ /µL and a C-reactive protein (CRP) of 29.2 mg/dL. No elevated levels of hepatobiliary system enzymes were observed. The patient underwent a contrast-enhanced CT again and revealed a 70 x 75 mm mass in the right lower lobe, which was suspected to be a lung abscess (Figure 4). Due to the history of a prior cholecystectomy of this patient, the diagnosis of postoperative intra-abdominal abscesses from dropped gallstones was made. We thought that the dropped gallstone was accompanied by infection and formed a subphrenic abscess, causing transphrenic pulmonary fistula and resulting in a pulmonary abscess. The dropped gallstone was present in the subphrenic abscess cavity.

**Figure 5:**
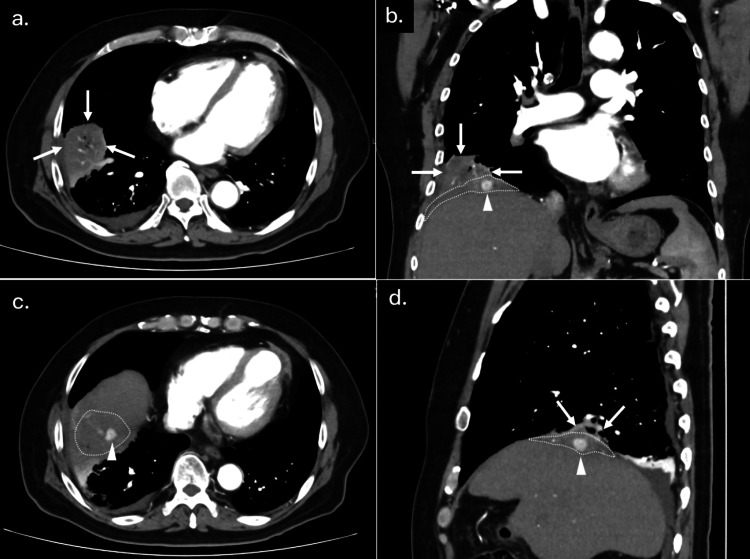
Following CT scan findings after antibiotic treatment (a-d) CT after antibiotic treatment showed the lung abscess (arrows), pneumonia, and subphrenic abscess (dotted areas) had improved. Arrowheads depict residual gallstone.

Since the patient had severe lung abscess and pneumonia, we planned a staged treatment strategy. At first, we treated him conservatively with an antibiotic to reduce the extent of surgery, then performed surgery after the inflammation was calmed and the abscess was reduced with antibiotics. He was treated with intravenous antibiotics (tazobactam/piperacillin) for 18 days, with a good response. A CT scan showed the lung abscess, pneumonia, and subphrenic abscess had improved (Figure 5).

**Figure 6:**
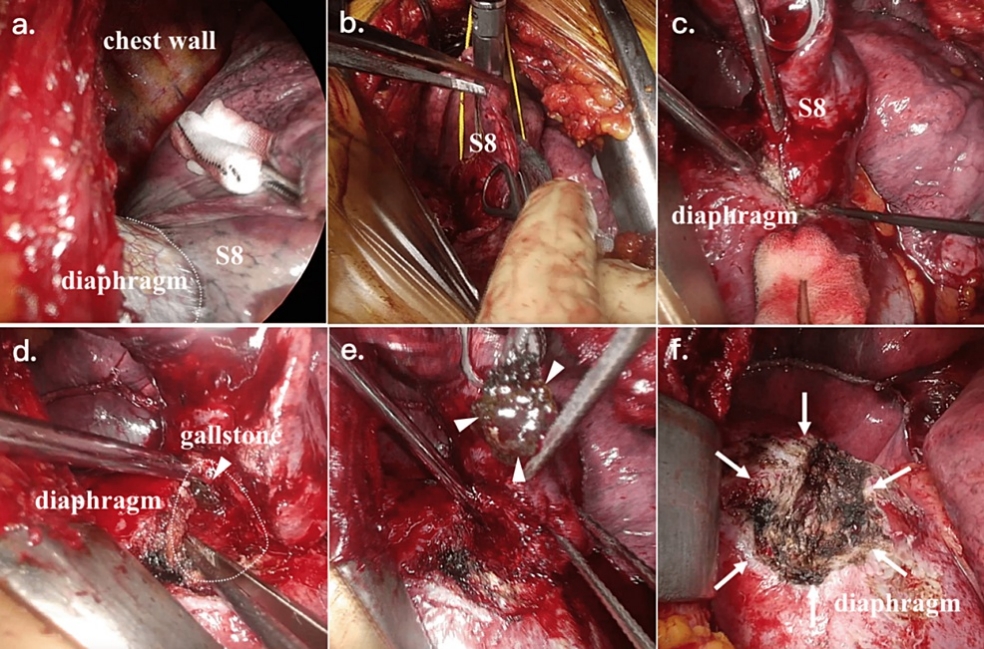
Intraoperative findings during partial resection of S8 in the lower lobe of the right lung, abscess drainage, and retrieval of the dropped gallstone (a) The dome of the diaphragm was adhered strongly with the S8 in the lower lobe of the right lung (dotted lines). However, the adhesions on the chest wall were mild. (b) S8 was taped and partially resected with a surgical stapler. (c) Adhesiolysis between S8 in the lower lobe of the right lung and diaphragm was performed. (d) The abscess cavity (dotted areas), which was extended into the subphrenic abscess cavity, was opened and a gallstone (arrowheads) approximately 1 cm in diameter was identified. (e) A gallstone was retrieved. (f) The diaphragm was exposed to 6 cm x 3 cm of muscular layer (arrows).

We performed partial resection of S8 in the lower lobe of the right lung, abscess drainage, and retrieval of the dropped gallstone (Figure 6 and Video 1). The chest was entered via a seventh intercostal space incision. The dome of the diaphragm, where inflammation spilled over from the abdomen, adhered strongly with the S8 in the lower lobe of the right lung. However, the adhesions on the chest wall were mild. After S8 was taped, S8 was partially resected with a surgical stapler. During the adhesiolysis between S8 and the dome of the diaphragm, the abscess cavity, which was extended into the subphrenic abscess cavity, was opened, and a gallstone about 1 cm in diameter was identified and retrieved along with pus. The pus cultures grew Klebsiella pneumonia, which was the same as the bile culture at the time of the cholecystectomy. The diaphragm was exposed to 6 cm x 3 cm of muscular layer, but due to adhesions with the liver, there was no intra-abdominal traffic, so suture closure of the diaphragm was not performed. A thoracic drain was placed, and the surgery was completed. Because the thoracic cavity was communicated with a subphrenic cavity, only a thoracic drain was placed in the current case. The gallbladder stone was a calcium bilirubinate stone, which was the same as the previous cholecystectomy. The resected specimen of the lung (S8) formed an abscess. The drain was removed on postoperative day eight, and the antibiotic was de-escalated to cefmetazole and administered for nine days postoperatively. The postoperative course was uneventful, and he was discharged on postoperative day 13.

**Video 1:** Intraoperative findings during partial resection of S8 in the lower lobe of the right lung, abscess drainage, and retrieval of the dropped gallstone

## Discussion

Laparoscopic cholecystectomy is a common surgical operation because laparoscopic surgery has the benefits of being less invasive and greatly reducing surgical pain and postoperative hospital stay. In recent years, the indication of laparoscopic cholecystectomy has been expanded to include the cases of severe inflammation, accidental perforation of the gallbladder, and spillage of gallstones into the abdominal cavity occasionally occur during laparoscopic cholecystectomy. The incidence of gallbladder perforation ranges from a reported rate of 10% to 40% of laparoscopic cholecystectomy, and spillage of gallstones occurred in 5.4% to 19% of the cases [1-4].

Schäfer et al. [3] reported 581 cases of spilled gallstones during 10,174 laparoscopic cholecystectomies, an occurrence rate of 5.7%. Thirty-four of these cases were converted to an open procedure in an attempt to remove lost gallstones, while of the remaining 547 patients, only eight (0.08%) developed postoperative complications requiring reoperation. A majority of dropped gallstones are retrieved intraoperatively. However, stones may become fragmented, inaccessible, or overlooked, leaving gallstones within the intra-abdominal cavity. Gallstones that have been spilled are usually clinically silent and considered harmless [5]. However, spilled gallstones may result in late complications of laparoscopic cholecystectomy, such as intra-abdominal abscesses, abdominal wall abscesses, intra-thoracic abscesses, retroperitoneal abscesses, and pelvic abscesses [6-8]. There is little research on thoracic complications caused by residual gallstones after laparoscopic cholecystectomy [9,10]. It is very rare for surgery to remove gallstones to leave behind a lung abscess that can't be treated.

Infectious bile juice, the number of residual stones, and pigmented calcium bilirubinate stones have been reported as risk factors for abscess formation due to dropped gallstones [11]. Development of abscesses secondary to dropped stones is demonstrated with an estimated average onset of four months to one-year post-cholecystectomy [12,13]. In the current case, multiple gallstones were dropped, and purulent bile was discharged from a highly inflamed gallbladder into the abdominal cavity, which were retrieved and washed out intraperitoneally as much as possible. However, the right subphrenic abscess appeared six months after the surgery. Furthermore, the patient had a complaint of fever, right chest pain, and hemoptysis, and CT revealed a lung abscess in the right lower lobe. We thought that the dropped gallstone was accompanied by infection and formed a subphrenic abscess, causing transphrenic pulmonary fistula and resulting in a pulmonary abscess.

The patient had severe lung abscess and pneumonia at the time of admission. Our operative strategy was to treat him conservatively with an antibiotic to avoid an expansion surgery, then perform surgery after the inflammation was calmed and the abscess was reduced with antibiotics. Fortunately, preoperative antibiotics were effective, the lung abscess shrank, and pneumonia improved. Then, we performed an operation. In the operative findings, fortunately, there was no evidence of empyema, and the adhesions in the thoracic cavity were not strong. This is because a subphrenic abscess caused a transphrenic pulmonary fistula and resulted in the lung abscess. However, the adhesion between the diaphragm and lung was so strong that a partial lung resection and a full-thickness resection of the adherent diaphragm needed to be performed. The dropped gallstone was present in the subphrenic abscess cavity, and it was a calcium bilirubinate stone. The pus cultures from the subphrenic abscess grew Klebsiella pneumonia, which was the same as the bile culture at the time of the cholecystectomy. In the current case, since the patient did not show any symptoms at all, we did not give any treatment at the time of the first CT, and we planned a short-term follow-up. However, one week after the first CT examination, the patient presented to the emergency department with complaints of hemoptysis. We think he may have avoided transphrenic pulmonary fistula and pulmonary resection if we started treatment just after the first CT.

Unlike other abdominal abscesses, which are treated with antibiotics and percutaneous drainage, removal of residual gallstones is imperative for an abscess associated with dropped gallstones. As the gallstone is a foreign body, if the gallstone is not removed, the abscess will recur, resulting in a prolonged course [5,12]. Although there is no established opinion about the duration of postoperative follow-up for cases of dropped gallstones, a survey reported that most surgeons (76.8%) follow up for about two years because of concerns about subsequent intra-abdominal infections [14]. In the current case, the follow-up of the patient was finished one month after the laparoscopic cholecystectomy because the patient had a good postoperative course. We should have followed up after discharge for a longer time.

## Conclusions

Laparoscopic cholecystectomy is widely performed for cholecystitis, and although not common, residual gallstones can cause late postoperative complications, such as not only intra-abdominal abscesses but also, although rare, intra-thoracic complications. To avoid injury to the gallbladder wall and spillage of gallstones during laparoscopic cholecystectomy, a careful operative procedure is important. However, if gallstones are dropped, it is important to attempt to retrieve all dropped gallstones during the operation as quickly as possible before they are lost. Furthermore, patients with dropped gallstones require long-term follow-up after discharge from the hospital.
